# Factors important for women who breastfeed in public: a content analysis of review data from FeedFinder

**DOI:** 10.1136/bmjopen-2016-011762

**Published:** 2016-10-24

**Authors:** Emma Simpson, Andrew Garbett, Rob Comber, Madeline Balaam

**Affiliations:** Open Lab, Newcastle University, Newcastle Upon Tyne, UK

**Keywords:** breastfeeding, public breastfeeding, PUBLIC HEALTH

## Abstract

**Objective:**

To examine how the breastfeeding experience is represented by users of FeedFinder (a mobile phone application for finding, reviewing and sharing places to breastfeed in public).

**Design:**

Content analysis using FeedFinder database.

**Setting:**

FeedFinder, UK, September 2013–June 2015.

**Methods:**

Reviews obtained through FeedFinder over a period of 21 months were systematically coded using a conventional content analysis approach, average review scores were calculated for the rating criteria in FeedFinder (comfort, hygiene, privacy, baby facilities) and review texts were analysed for sentiment. We used data from Foursquare to describe the type of venues visited and cross-referenced the location of venues with the Indices of Multiple Deprivation.

**Results:**

A total of 1757 reviews were analysed. Of all the reviews obtained, 80% of those were classified as positive, 15.4% were classified as neutral and 4.3% were classified as negative. Important factors that were discussed by women include facilities, service, level of privacy available and qualities of a venue. The majority of venues were classified as cafes (26.4%), shops (24.4%) and pubs (13.4%). Data on IMD were available for 1229 venues mapped within FeedFinder, 23% were located within the most deprived quintile and 16% were located in the least deprived quintile.

**Conclusions:**

Women create content that is positive and informative when describing their breastfeeding experience in public. Public health bodies and business owners have the potential to use the data from FeedFinder to impact on service provision. Further work is needed to explore the demographic differences that may help to tailor public health interventions aimed at increasing breastfeeding rates in the UK.

**Strengths and limitations of this study**

- To the best of our knowledge, this is the largest and most recent analysis of women’s experiences of breastfeeding in public.
- The use of a mobile phone application (FeedFinder) is a novel data collection method and reduces social desirability bias.
- We are unable to report on the sociodemographic profiles of the users; therefore, we cannot suggest that this is a fully representative sample of the breastfeeding community in the UK.

## Introduction

The latest report from Public Health England (PHE) details that a third of breastfeeding women choose not to breastfeed in public, and one in ten new mothers abstain from breastfeeding altogether due to the fear of breastfeeding in public.^1^ Additionally, the Infant Feeding Survey (2010) reported nearly half of breastfeeding women (47%) encountered problems when trying to find a suitable place to breastfeed in public and 11% had been stopped or made to feel uncomfortable when doing so.^2^ Despite these figures, women are legally protected to breastfeed publicly (up to 26 weeks) by the Equality Act 2010 and any public service providers (eg, cafes, libraries, public transport, etc) have a legal obligation to protect breastfeeding women from discrimination.^3^

Further qualitative research^4–12^ highlights similar concerns to these reports, illustrating that a women's comfort to feed in public is related to the perceived social, cultural and public values around breastfeeding. It has been identified previously that such values may be derived from seeing very few women breastfeeding in public in the UK,^6^ ^13^ alongside a lack of breastfeeding support in TV, print and social media.^14–16^ With regular high-profile media stories often detailing women's harassment for public breastfeeding,^3^ ^17^ ^18^ this can lead to the impression that the UK public are not breastfeeding friendly. Attitudes towards breastfeeding from society can be particularly influential and there are clear differences between different socioeconomic groups with younger, less affluent White females having the lowest uptake of breastfeeding.^2^ ^19^ Women often have to negotiate decisions to breastfeed among multiple contradictory sociocultural and discursive practices, particularly when in public. Breastfeeding is considered an ideological position of ‘good mothering’ but getting it ‘right’ when nursing outside of the home exerts further pressure on women. The idea of success is fostered through the art of being discreet and unnoticeable but (ideally) in private, while not adhering to such expectations somehow positions breastfeeding as morally wrong.^10–12^ It is considered that increasing exposure to breastfeeding will augment its normalcy and acceptance, which bears significance for those socioeconomic groups where breastfeeding is less common and among those who breastfeed, but rarely in public.^2^ ^20–24^

Despite the known obstacles, breastfeeding rates overall in the UK have increased in recent years, though still fall below NHS and WHO recommendations.^2^ Increasing breastfeeding rates remains a public health priority, and ensuring women have a good public breastfeeding experience is a key part of this agenda.^25^ The more recent *Lancet* series on breastfeeding^24^ ^26^ further details the need to increase breastfeeding rates worldwide and places emphasis on society as a whole to take responsibility. Moving forward, demands fostering positive societal attitudes towards breastfeeding; reinforcing a breastfeeding culture and overcoming restrictions of breastfeeding in public.

Our study was designed to describe women's recent experiences and preferences for public breastfeeding facilities in the UK. We systematically analysed 1757 free-text reviews of women's breastfeeding experiences as reported using FeedFinder.^27^ To complement the findings of the Infant Feeding Survey and PHE, we provide an analysis of women's' experiences that are current, likely to be in-the-moment and venue specific. This study contributes to a wider cross-disciplinary research agenda looking at the potential impact of social computing on public health ongoing at Open Lab, Newcastle University.

### FeedFinder

**Figure 1:**
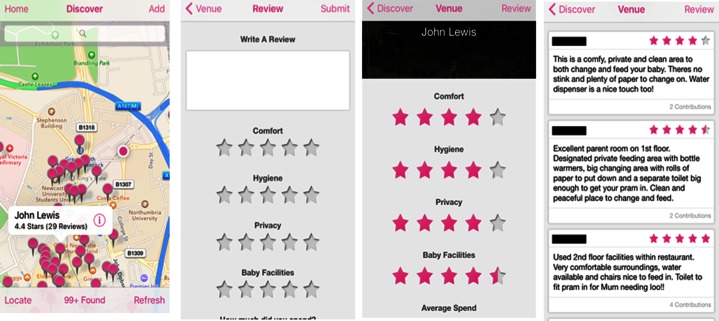
FeedFinder mobile app. (1) Location of user and surrounding venues mapped. Selecting a venue (pink pin) shows the name of venue, average star rating overall (out of 5) and number of reviews left by other users. (2) Submitting a review, free text entry box and rating categories out of 5 stars and sliding scale for average spend. (3) Opening a venue shows the average star rating for the four categories cumulated from other users. (4) Text reviews are listed under the star rating for each venue.

FeedFinder was codesigned with breastfeeding women and developed by a multidisciplinary research team at Open Lab.^27^ The app was developed with the intention of creating a supportive health technology for women to make the decision to breastfeed in public. It is a mobile application, available for free on iOS and Android, and allows users to find and review venues for how breastfeeding friendly they are on a map. The application makes use of the global positioning sensor (GPS) to centralise a user's location and present nearby community-added venues. Users are able to add a new venue and rate it based on 5 measures; comfort, hygiene, privacy, baby facilities and average spend and also leave an additional text review, as shown in figure 1. Formative design workshops with breastfeeding mothers identified these measures as key for contributing to a positive breastfeeding experience. Launching in July 2013, FeedFinder has been running at time of publication for over 38 months and has seen an uptake of almost 10 000 users worldwide. For the context of this research, we present findings on the users within the UK only.

The aim of the present study was to systematically analyse the review content provided by breastfeeding women on FeedFinder and ultimately gain an insight into the factors that are considered important when breastfeeding in public.

## Methods

### Consent

All users who download FeedFinder are informed through the terms and conditions that FeedFinder is part of an ongoing research project. By accepting the terms and conditions, users’ consent to their information is used in this research. All reviews are anonymised for the purposes of reporting.

### Study design

We conducted a conventional (inductive) content analysis of the review data obtained through FeedFinder.^28^ The purpose of the study was to capture and describe factors important to women who use FeedFinder when breastfeeding in public; therefore, an inductive approach was agreed to be the most appropriate. Initial codes were derived directly from the data. A team of four researchers were involved in the process of coding and analysis; ensuring credibility through peer-to-peer debriefing and member checking of the codes and categories constructed from the data.^28^ Adding to the content analysis of the text reviews, we also summarised the average and overall rating of the measures collected through FeedFinder: comfort, hygiene, privacy, baby facilities and calculating the average spend. We also reported on the type of venues reviewed with data available from Foursquare (https://foursquare.com/—a location based social networking site and application that uses crowdsourced data to identify buildings, businesses and attractions),^29^ for example, cafes, pubs, restaurants, public health service providers, public spaces and miscellaneous. The Indices of Multiple Deprivation^30^ were cross-referenced with the location of venues mapped.

#### Coding and analysis

All reviews left on FeedFinder within the UK between September 2013 and June 2015 were collected. Three independent researchers openly coded the same random selection of 5% of the data set (n=90) and reconvened to discuss the commonalities and intercoder reliability. The random 5% was selected before any reviews were omitted from the total analysis. Each researcher then coded an additional 5% applying the codes that had been generated in the previous selection and adding to it any new ones that surfaced. The remaining 1595 reviews were coded by two of the researchers. A systematic procedure was in place to discuss constructed codes and categories in a feedback loop with the remaining research team. A further sentiment analysis was carried out on the reviews—coding each review as positive, negative or neutral based on the descriptive words and context of the review. Again, this was a systematic procedure with the research team and any queries were discussed until an agreement could be reached.

## Results

In total, 1869 reviews were obtained. Of these 1869 reviews, 112 were removed due to duplicates, empty text boxes and reviews not from the UK.

The remaining 1757 text reviews were analysed from 1416 individual venues and 783 contributing reviewers.

The analysis of the reviews generated 63 separate codes that were then categorised into 14 separate groups, see table 1. Altogether, the 1757 reviews were assigned 6540 individual codes. The length of reviews varied widely across the data set from concise descriptive accounts to a much more detailed explanation of the user's experience while breastfeeding at a venue; the average character count of the text reviews was 136 (±90) with a minimum of 2 and a maximum of 400 characters (∼80 words). Expectedly, the amount of codes for each review varied widely ranging from 1 to 13. Illustrative examples of the types of reviews are shown in table 2. Further analysis using available Foursquare data informed us of the types of venues that women reviewed in FeedFinder. However, Foursquare data were available only for 41% of venues mapped within the app.

**Table 1:** Categories derived from analysis of data

Categories	Total number of codes	% of reviews	% of reviews identified as negative
Facilities	862	49.00	0.55
Service	836	47.58	0.26
Privacy	723	41.15	0.70
Qualities	690	39.27	0.15
Furniture	619	35.23	0.03
Built environment	526	29.94	0.08
Visit	365	20.77	0.17
Food and drink	359	20.43	0.14
Misc.	342	19.46	0.34
Inclusivity	331	18.84	0.41
Support	324	18.44	0.05
Experiential	260	14.80	0.21
Atmosphere	194	11.04	0.05
Entertainment	130	7.40	0.11
total	6560		

The total number of codes assigned from each category and % and what proportion of the reviews were negative.

**Table 2:** Illustrative examples of reviews with sentiment and codes assigned

Sentiment of review	Examples of review text	Codes assigned
Positive	“Lovely cafe, staff have always been welcoming and have often brought drinks to our table when feeding. One member even offered to cut my food up when I was with my new-born. Lovely changing facility too.”	Welcoming, staff, hospitality, changing, anecdote
	“Excellent facilities. Discreet corners in cafe and a designated feeding room that is separate from changing room. Always felt comfortable feeding in cafe. Staff very helpful, carried tray to table. Also free Wi-Fi to keep you entertained during those lengthy feeds :-)”	Facilities, designated area, privacy, changing, comfortable, revisit, staff, hospitality, Wi-Fi,
Negative	“Feeding area is disgusting and unsanitary. As well as being in an awkward place. Will not be using it again. Was absolutely appalled.”	Cleanliness, designated area
	“Appalling! Was told that I was no longer allowed to breastfeed my daughter (sibling of son who attended nursery) unless I covered up or fed in a different room away from children.”	Stigma, exclusion
Neutral	“Bosom Buddies Thursday 9.30–11.30″	Classes
	“Room at the back for feeding and changing.”	Designated area, way finding, changing

### Coding results

Almost half of the reviews contained at least one reference to facilities and services, 49.0% and 47.58%, respectively. The facilities category covered any codes relating to a range of amenities, including changing, bottle feeding facilities, plug sockets and Wi-Fi availability. Services included explicitly mentioning staff, hospitality and a venue being welcoming of breastfeeding. Of those reviews that mentioned staff and hospitality, there was particular emphasis on the ways in which the staff were helpful, that is, providing table service when they usually have an order and receive at the counter style procedure. Over 40% of reviews contained information on the level of privacy available in a venue. Reviews were coded for privacy if a review mentioned a designated area for breastfeeding (n=240), if there was expectation to feed in a disabled toilet (exclusion from public space) (n=9), or the reviewer simply offered an opinion on the level of privacy available (n=474). Qualities of a venue were also frequently mentioned in reviews, 39.27%, including reference to how relaxing, comfortable, clean the venues were (although this may have been a result of the five categories women are asked to rate a venue by), but also other qualities including the lighting, music and temperature. In most cases, comfort was often described alongside the type of furniture provided (35.23% of reviews). The built environment included information on access (with a pram), space, outdoor facilities and way-finding within a venue which were included in almost 30% of reviews.

Almost 15% of reviews included an experiential account of breastfeeding in the public space including anecdotes, reference to stigma and whether they stated to be a new/first time mother, or first public feed. In addition, only 83 reviews contained reference to social stigma associated with breastfeeding in public, including review text such as “…no-one batted an eyelid” and “…you might get some odd looks or comments from some customers who are not used to seeing babies being fed”. Inclusivity was described as being baby/toddler and child friendly. Table 1 also indicates which of the categories were more prominent in the negatively coded reviews and privacy was mentioned most; 12 reviews in total. Inclusivity in the reviews with negative sentiment was mentioned in seven of the reviews. For example, women expressed disgust with expectations to nurse an infant in a baby changing toilet, with comments such as “A chair in a nappy changing.  … Wedged between toilet and nappy bins. Pretty disgusting and marked as a designated feeding area. I wouldn’t feed an animal in there…”.

### Sentiment analysis

Sentiment of each free text review was determined based on the type of descriptive words used and the overall context of the review. Each of the text reviews were expressed as being either positive, negative or neutral. Some reviews contained positive and negative elements and were therefore coded as neutral. The majority of reviews were considered positive—80.3% (n=1410) positive, 4.1% (n=72) negative and 15.7% (n=275) neutral. Further analysis of the reviews indicated that, of the 74 negatively coded reviews, only three of those were because someone in the venue asked them to stop breastfeeding or made them feel uncomfortable doing so. In total, only 0.2% of the data set experienced a negative reaction from the public. Examples of the types of reviews are illustrated in table 2.

### FeedFinder criteria ratings

Users are able to rate venues (out of five) based on four different measures that potentially meet their breastfeeding needs; comfort, hygiene, privacy and baby facilities as well as inputting their average spend. We can consider that an average rating would be 3* out of 5, poor <3 and excellent >3. The average rating for each measure for all reviews obtained is shown in table 3. Each measure overall had an average >3 of 5. Hygiene was rated highest at 3.9/5 overall and privacy was rated lowest 3.2/5.

**Table 3:** Average star rating for each of the four measures collected through FeedFinder for all venues reviewed by users and average spend

	Comfort	Hygiene	Privacy	Baby facilities	Average spend (£)
Average (out of 5)	3.7	3.9	3.2	3.3	5.33

The average spend overall was £5.33 from 998 individual reviews, which indicated a spend more than £0. The remaining reviews (n=759) reported no spend.

### Types of venues reviewed

We are able to report the types of venues that FeedFinder users map and review; however, Foursquare data were available only for 583 venues (41%). Foursquare provides a category for each type of venue and we grouped some of the venue categories together for reporting ease. For example, ‘Thai restaurant’, ‘Asian restaurant’, ‘fast food restaurant’ were all categorised under ‘restaurant’; ‘doctors’, ‘dentist’ and ‘pharmacy’ were all categorised under ‘public health services’; ‘public spaces’ included ‘parks’, ‘farm’, ‘library’; and ‘miscellaneous’ included those that could not be easily categorised such as ‘courthouse’, ‘office’. Cafes are the most popular type of venue where FeedFinder users experienced breastfeeding in public (26.2%), followed by shops (24.4%), pubs (13.4%), public spaces (8.7%), public health services (3.8%) and miscellaneous (2.7%).

### Indices of Multiple Deprivation and venue location

We cross-referenced the location of venues with the Indices of Multiple Deprivation (IMD).^30^ Based on the Lower-layer Super Output Areas in England (LSOA—(areas/neighbourhoods with populations ≤1500)) IMD scores are ranked from 1 (most deprived) to 32 844 (least deprived) and we describe in relation to quintiles (20%). We have data available for 1229 venues in England (87.9% of the full data set). Our analysis shows that 23% of venues reviewed were in the most deprived quintile and 16% were located in the least deprived quintile. The remaining venues were distributed similarly across the three middle quintiles; 2nd—19%, 3rd—22% and 4th—20%.

## Discussion

FeedFinder is an example of a user-led public health intervention and we can demonstrate through our data analysis the type of content women create for each other is informative and predominantly positive. In the free text comments, users of FeedFinder reported on a number of different aspects relating to their breastfeeding experience, but mainly facilities available within a venue, service and hospitality from staff, qualities (mainly comfort) and level of privacy available. Our data analysis complements the findings from the Infant Feeding Survey (IFS) which indicated an expectation of ‘facilities’ being provided in shopping centres and restaurants with 90% and 78% of women agreeing, respectively, that facilities should be provided.^2^ However, the type of facilities is not specified in the report. In our data, women often reported on the changing facilities available within a venue but also informed other FeedFinder users on the availability of alternative feeding facilities (bottle warmer, microwave), plug sockets and Wi-Fi.

A significant barrier to the uptake of breastfeeding among women is the fear of having to breastfeed outside of the home,^1^ ^4^ with the media often reinforcing those anxieties by reporting on mothers' bad experiences.^16–18^ Other literature around the conversation of breastfeeding in public define success as being naturally discreet, unnoticeable and ideally in private to avoid unwarranted negativity from the public.^10–12^ Our analysis further reinforces this ideological position on breastfeeding in public with over 40% of our reviews referencing ‘privacy’, which also covered being able to feed ‘discreetly’ and locating a designated breastfeeding area. Women often provided advice on ‘nooks’ and hidden corners where women can feed privately without disturbing the public. Further analysis indicated that in the reviews containing negative sentiment, privacy was mentioned most often.

Our analysis indicates that of those women who use FeedFinder when breastfeeding in public, 80.3% of those experiences were coded as positive, 15.5% were coded as neutral and 4.2% were coded as negative. Further to this, of those who we classified as negative, only 3 of the 74 negatively coded reviews explicitly detailed that someone in the venue asked them to stop breastfeeding or made them feel uncomfortable doing so. In total then, only 0.2% of the data set overall experienced a negative reaction from the public. Compared with the IFS which reported 11% of women surveyed had been asked to stop breastfeeding in a public place, our results indicate a much lower proportion of women experiencing any discrimination towards them. The different methodological approaches between the IFS and FeedFinder data might explain some of the differences in results. First, IFS survey participants are asked explicitly whether they have experienced negativity when breastfeeding in public, whereas users of FeedFinder are presented with a free text box and no guidance of what to include in their review. Although now discontinued, the main aim of the IFS was to provide estimates on the incidence, prevalence and duration of breastfeeding and other feeding practices to inform the academic community and FeedFinder data are provided for women to use when seeking out places to breastfeed. Compared with the IFS traditional style survey, FeedFinder can be considered a tool of *ecological momentary assessment* (EMA). An EMA can be described as a tool for collecting real-time data in real-world settings while minimising retrospective bias.^31^ The use of mobile technology as a platform for EMA is increasingly being recognised in health research.^32–34^ Women using FeedFinder are reporting on their experience in a specific location at a specific time point whereas completing the IFS requires the women to recall their experiences at three different stages. This could potentially introduce recall bias. Further supporting this theory, research into memory recall has demonstrated that negative elements of an event are more likely to be remembered compared to positive and neutral elements.^35^ This is particularly salient when considering emotional stimuli connected to an event. Breastfeeding is as much about emotions as it is about the physical transfer of milk from mother to infant. The emotional connections attached to being told to stop breastfeeding in public could potentially permeate the memories of breastfeeding in public for women.

We must also acknowledge that unlike the IFS, the demographic and sociocultural characteristics of women using FeedFinder are currently unknown. It could be that women using FeedFinder possess characteristics that might make public breastfeeding easier such as maternal age, ethnicity, level of deprivation, education, profession, attending breastfeeding support groups and personal or familial breastfeeding experience.^2^ ^20^ ^23^

It is possible, however, that women using FeedFinder have under-reported their negative experiences or that the attitude to breastfeeding in public is changing as awareness is increased with interventions such as.^36^ ^37^ Though, as a social sensing tool, we view FeedFinder as playing a supportive role and some women may tend to report more on the positive experiences rather than the negative.

In reference to the stigma around breastfeeding in public, women mentioned instances where “no one batted an eyelid” and they “never had any bad comments” and of the total number of reviews, only 83 instances referred to an expected negative response from the public. This suggests a small proportion of the breastfeeding users of FeedFinder themselves are informing and reassuring each other that although a negative reaction might be expected, it very rarely happens. The challenge here is helping new mothers to find out about the positive experiences that other breastfeeding women describe.

From our textual data analysis we can conclude that in FeedFinder, the breastfeeding users produce informative content for each other about locations for public breastfeeding, suggesting that they have a feeling of responsibility in supporting other new breastfeeding women as their motivation for producing content within the application. FeedFinder could be an alternative means for collecting information on breastfeeding practices in public and help to inform the academic community and those working in practise.

### Types of venues and IMD

Interestingly more venues were mapped in the most deprived 20% of England overall compared with the other quintiles. There could be a number of reasons for this, including the IMD score of urbanised, city centre locations tending to be areas considered most deprived. However, we are unaware at this stage if the venues are mapped from women residing in these areas or if they are women who have travelled from outer city suburbs (generally considered less deprived). We do know that typically, breastfeeding rates are lowest in areas of high deprivation. Future research would seek to understand these differences seen in the location of venues mapped while further developing the app to gather sociodemographic information from users which would help foster these understandings.

### Using the data in practice

Public health professionals could use the FeedFinder review data to shape their interventions around community support of breastfeeding. Identifying low-rated venues or negative reviews in local areas would help public health professionals use a more targeted approach to increasing support while using their available resources. They could use this information to offer training and advice to businesses and use FeedFinder to track the progress by monitoring further reviews post intervention. In addition, businesses have a vested interest in obtaining excellent scores in online resources such as TripAdvisor and for food hygiene ratings. Failing to obtain a high standard of reviews can encourage a business to improve the quality of service they provide. With FeedFinder then, businesses could monitor the information provided by breastfeeding women to ensure they provide a high standard of breastfeeding support while attracting new breastfeeding mothers (and therefore more business) into their venue. They can also use these data to find out what kind of support women want when breastfeeding in their venue. Reviews can act as a critical view of what works and what does not in relation to breastfeeding support but they can also act as a marketing tool by spreading information on good experiences, particularly when a venue gets something right.^38^

### Strengths and potential limitations

We have presented an analysis of the free text comments provided by FeedFinder users. To the best of our knowledge, this is the largest source of information regarding women's experiences in public with such detail, adding further knowledge to the IFS which indicated an expectation of facilities in restaurants and shopping centres. Additionally, the use of the mobile application to record experiences has the potential to eliminate social desirability bias that might be experienced in an interview situation.^39^ ^40^

Although we have found our users have an overwhelmingly positive experience when breastfeeding in public, this may not be representative of all women's experiences of breastfeeding in the UK. We are unable to report on the sociodemographic profiles of the users; therefore, we cannot suggest that this is a fully representative sample of the breastfeeding community in the UK. Moreover, the number of users who leave a review is a relatively small percentage of the total users who download FeedFinder. Additional work is needed to explore the different types of users of FeedFinder, that is, those who leave reviews compared with those who seek out and explore the places on the map with further analysis on their motivations of use. We reported a low number of negative reviews but we acknowledge the possibility that some women may choose not to report on their negative experiences.

### Conclusion

We can conclude from this research that the content women provide for each other is informative and positive. Facilities, hospitality, level of privacy available appear to be the most important factors for enabling a woman to breastfeed comfortably outside of the home (according to our FeedFinder users). Further work is needed to explore the demographic data of the FeedFinder users and explore whether certain factors are significant among different social groups or communities. For example, is privacy more important in areas of high deprivation? This could better inform public health services in shaping successful interventions to increase support of breastfeeding within the UK.
